# Focus Group Study Exploring Factors Related to Frequent Sickness Absence

**DOI:** 10.1371/journal.pone.0148647

**Published:** 2016-02-12

**Authors:** Annette Notenbomer, Corné A. M. Roelen, Willem van Rhenen, Johan W. Groothoff

**Affiliations:** 1 ArboNed Occupational Health Service, Utrecht, The Netherlands; 2 Department of Health Sciences, division Community and Occupational Medicine, University Medical Center Groningen, University of Groningen, Groningen, The Netherlands; 3 Department of Epidemiology and Biostatistics, VU Medical Center, VU University, Amsterdam, The Netherlands; 4 Center for Human Resource, Organization and Management Effectiveness, Business University Nyenrode, Breukelen, The Netherlands; Catholic University of Sacred Heart of Rome, ITALY

## Abstract

**Introduction:**

Research investigating frequent sickness absence (3 or more episodes per year) is scarce and qualitative research from the perspective of frequent absentees themselves is lacking. The aim of the current study is to explore awareness, determinants of and solutions to frequent sickness absence from the perspective of frequent absentees themselves.

**Methods:**

We performed a qualitative study of 3 focus group discussions involving a total of 15 frequent absentees. Focus group discussions were audiotaped and transcribed verbatim. Results were analyzed with the Graneheim method using the Job Demands Resources (JD–R) model as theoretical framework.

**Results:**

Many participants were not aware of their frequent sickness absence and the risk of future long-term sickness absence. As determinants, participants mentioned job demands, job resources, home demands, poor health, chronic illness, unhealthy lifestyles, and diminished feeling of responsibility to attend work in cases of low job resources. Managing these factors and improving communication (skills) were regarded as solutions to reduce frequent sickness absence.

**Conclusions:**

The JD–R model provided a framework for determinants of and solutions to frequent sickness absence. Additional determinants were poor health, chronic illness, unhealthy lifestyles, and diminished feeling of responsibility to attend work in cases of low job resources. Frequent sickness absence should be regarded as a signal that something is wrong. Managers, supervisors, and occupational health care providers should advise and support frequent absentees to accommodate job demands, increase both job and personal resources, and improve health rather than express disapproval of frequent sickness absence and apply pressure regarding work attendance.

## Introduction

Long-term sickness absence and return to work are widely researched topics in occupational health care. By contrast, frequent sickness absence has scarcely been investigated. Long-term sickness absence is associated with severe illness or medical conditions that fail to recover [1]. Frequent absence, on the other hand, is often considered by researchers to be a psychological phenomenon, driven by motivational or behavioral processes [2–4], although this view is not conclusive [5,6]. Frequent sickness absence disturbs work schedules, affects social relations at the workplace, and may deepen feelings of distrust and blame among colleagues [7]. In addition, frequent sickness absence may also be a risk factor for long-term sickness absence and work disability [8,9]. Koopmans et al. (2008) reported that 50% of employees who had four or more sickness absence episodes with a duration <6 weeks in a baseline year experienced long-term (≥6 weeks) sickness absence in the following four years [8]. Other studies have defined frequent sickness absence as three or more sickness absence episodes in a year [10,11].

Minor morbidities such as upper respiratory infections and gastro-intestinal problems are the most commonly self-reported diagnoses among frequent absentees [12]. The mild nature of these complaints leaves an employee a certain latitude to decide whether to call in sick or go to work. Johansson and Lundberg reported that going to work when ill depends on attendance requirements. In women, sickness attendance was also associated with low adjustment latitude, defined as the possibility to accommodate work to health complaints [13]. Attendance requirements and adjustment latitude depend on psychosocial working conditions. There is a large body of evidence that psychosocial working conditions are associated with sickness absence.

In the Netherlands, the Job Demands–Resources (JD–R) model is widely used (in organizational psychology and occupational health) to describe psychosocial working conditions [14]. The JD–R model is an alternative of Karasek’s Job Demands Control model and more comprehensively captures the factors that play a role in employee well-being. The model originally contained only issues related to job demands (e.g. workload, emotional demand and work-home interference) and job resources (e.g. social support from a manager or colleagues, autonomy, opportunities to learn and feedback). Home demands and resources were later added. Burdensome domestic roles and stressful life-events are examples of home demands and support from family and friends are examples of home resources [15]. Personal resources such as self-efficacy, self-esteem and optimism have also been added to the model [16]. A study by Schaufeli and colleagues showed that increased job demands were associated with longer duration of sickness absence, while decreased job resources were associated with a higher frequency of sickness absence [4]. High home demands increase sickness absence duration and frequency, whereas home resources decrease these factors [6]. Over time, personal resources reinforce job resources [17] and buffer the adverse health effects of job demands [18]. Job resources drive the motivational process. Job resources can satisfy psychological needs such as autonomy and relatedness, consequently enhancing motivation for work [4, 19, 20].

The aim of the present study is to explore frequent sickness absence from the employee’s perspective by using a focus group design. We address the following issues:

- awareness of being a frequent absentee and having a risk of long-term sickness absence;
- determinants of frequent sickness absence;.
- solutions to reduce frequent sickness absence.

Ideas and beliefs of frequent absentees may provide clues for further research and interventions aimed at reducing the frequency of sickness absence.

## Methods

For this qualitative study, we used focus groups to gather information and share perspectives, without the prerequisite to reach consensus [21]. Three focus groups met in April 2012 made up of employees who had had three or more sickness absence episodes in the previous year, irrespective of duration of or reason for the sickness absence. The Medical Ethical Committee of the University Medical Center Groningen granted ethical clearance for this focus group study (reference METc2012.041). All participants provided informed written consent to participate in the study.

### Participants

For this study, we contacted 16 large (i.e. staffing more than 100 employees) organizations in the Dutch province Friesland, all clients of ArboNed, a large Dutch occupational health care provider. Eleven companies, staffing a total of 3399 employees agreed to participate. In the participating companies, 309 employees (9%) were frequent absentees in the sense that they had had three or more sickness absence episodes in the past year. We assigned random numbers to these frequent absentees, using random number tables, and ranked them by increasing number. To ensure employee privacy and facilitate open group discussions, from any particular company we included only one employee in a focus group. The first researcher (AN) phoned the employees by order of rank to invite them for the study. Employees were contacted until 21 agreed to participate.

Five participants cancelled just before the focus group meeting took place because of medical treatment, family reasons and fear of talking about private matters; one participant did not show up. The remaining 15 employees participated in one of three focus groups (N = 6, N = 4, N = 5).

### Focus group method

The groups were led by the same independent moderator, a psychologist experienced in groups discussions in corporate settings. None of the participants had had contact with the moderator before the meeting.

The focus groups met in a conference room at ArboNed. Participants signed informed consent forms before the group started. They only received reimbursement for travel costs. The moderator introduced the topic of frequent sickness absence and explained that he wanted to learn as much about the views and opinions of the participants as possible, all of whom had been absent three or more times in the previous year. He then started an introduction round, asking each participant to tell something about himself and his work, thereby getting them used to speaking up in the group.

During the focus group discussion, the moderator adhered to the structured interview schedule (Table 1). First he asked the participants about their awareness of being frequent absentees and their considerations about reporting sick. The key questions addressed the determinants of frequent sickness absence, solutions to reduce frequent sickness absence and awareness of the risk of long-term sickness absence. Open questions were used to get unprejudiced information and followed up by additional cues to adhere to the interview schedule. We took these cues from the JD-R model, used as the framework for our study, but we also allowed room for other themes not yet included in the JD-R model. To prevent intellectualized answers [21], we asked participants not only to reflect on reasons for their own pattern of frequent sickness absence, but also 1) to project their own feelings when asked to give their general reasons for frequent sickness absence, 2) to explain what they felt was needed to influence frequent sickness absence and 3) to express explicitly what they needed from others. On top of that three additional statements were used to trigger further responses (Table 1). The question on awareness also provided clues to the underlying processes leading to frequent sickness absence. The moderator observed subjects’ level of participation and invited those who did not spontaneously join in to speak up, thereby ensuring that everyone had a say in the discussions. Each focus group meeting lasted two hours with a short break.

**Table 1:** Introduction, key questions and statements in chronological order.

**Introduction**	
We would like to get your help as an expert: you all called-in sick at least 3 times last year.	
**Key questions**	**Probe**
What is the reason for people to be relatively often absent, and how is this for you?	Motivation, lifestyle, behavior, self-efficacy, upbringing, private issues, work/private interference, work situation, culture, health, adequate medical diagnostics/therapy.
How can people influence the frequency of sickness absence, and what do you need?	Communication with manager about working conditions, lifestyle, balance, smart(er) working (body/mind), visit to GP or occupational physician.
What do people need from others to prevent calling in sick, and what do you need?	Support at work from colleagues, manager, accommodated work, support at home, adequate medical help.
**Statements**	
People who are frequently absent take insufficient care of their health	Feeling/intuitive opinion. In case of sufficient attention for own health: is this adequate attention on health, health-promoting (work posture, self-management, health management, patient compliance, optimal medication.
I would like to be on sick- leave less frequently	Why?
I would like help to get be in control of the frequency of my sickness absence. And what would you say if we changed the question into: I would like to be more in control of my health?	How? For example online tools, feedback, advices, occupational physician, other?
**Key question**	
Scientific research has proven that 50% of frequent absentees will become long-term absentees within four years. How do you relate to that?	

### Data analysis

The focus group discussions were audio-taped and transcribed verbatim. The first researcher made field notes and after each session checked notes with the moderator. We analyzed the qualitative data using the Graneheim method [22]. First we identified meaning units from the fully transcribed focus groups and put them into the analysis. Then, three researchers (AN, CR and JWG) independently translated the meaning units into condensed meaning units. We then compared the condensed meaning units and discussed differences to reach consensus. Next we translated the condensed meaning units into codes. During this process, we frequently went back to the transcripts and sometimes the audio tapes, to ensure that the themes reflected actual data instead of researchers’ interpretation. We used the expanded JD–R model as a theoretical framework to structure the codes into themes. Codes that did not fit into the expanded JD–R model were acknowledged as separate themes.

## Results

The participants (10 men, 5 women) had a mean age of 48.6 years and were permanently employed in various jobs ranging from production workers to professionals (e.g. technicians and teachers).

### Awareness

Before the study, many participants were not aware that they were frequent absentees. Some were not interested in how often they reported sick, while others had been made aware of their frequent sickness absences by their manager or colleagues. Most participants seemed to feel a need to explain their frequent sickness absence and when asked about their awareness of it they spontaneously mentioned medical reasons or complaints.

The participants could hardly believe that frequent sickness absence posed a risk for future long-term sickness absence, even though some of them were already long-term sick-listed. The long-term sick-listed participants did not consider themselves as being on sick-leave when they were (partially) working in accommodated tasks: *“I have never thought about long-term absence*, *I already work six hours a day*” (note author: in accommodated tasks). Most participants believed that they would not become long-term absentees within the next few years: *“I can’t imagine myself to be on long-term sick-leave in the future*, *however*, *I can imagine this happening to people who overexert themselves for too long”*. The focus group discussions stimulated reflection on the part of some participants, whose previous unawareness was replaced with new insights: *“I can’t imagine myself calling-in sick for a long period of time within the next four years*, *however*, *we have a lot in common*, *we all want to do a lot of things*, *and we have to be aware where the boundaries are”*. One participant regarded the information on the relationship between frequent and long-term sickness absence as a signal: *“frequent sickness absence is a signal for me to take action; I want to avoid belonging to those who become long-term sickness absentees”*.

### Determinants of frequent sickness absence

The participants mentioned several job demands, such as work pressure *(“we do the same work with fewer colleagues”*, *“work pressure*, *preventing functioning well in your job”*) and a misfit between job and person *(“when your work doesn’t fit*, *you experience work stress instead of work pressure”*) as reasons for frequent sickness absence. One participant could no longer deal with irregular work shifts: *“it becomes more difficult (over the years) to have different working rhythms over the course of a few days”*. One participant with migraine mentioned that the workplace was too warm. For some participants, job demands exceeded their capacity to work, leading to not feeling well and problems with work functioning, which in turn led to frequent sickness absence: *“work has become more burdensome*, *it starts pressing on you*. *When it presses too much*, *you get flu-like symptoms”*, *“you try and try*. *At a certain point in time your body stops functioning and it is over”*, *“I have a very intensive job*, *leading to mental overload*, *I then have the tendency to take a break from work”*, *“I am not sure how long I can do this*. *The work is very intensive*. *Last summer my brain was in overdrive for 3 quarters of an hour after I arrived home*, *before I started to calm down*. *My body didn’t want to calm down*. *I cannot manage (my job) any more”*, *“too much external pressure*, *making it impossible for me to function any more”*. In other cases a chronic disease reduced the capacity to work, leading to frequent sickness absence when job demands were not adjusted to the lower work capacity. A participant with arthrosis in combination with a physically demanding job said: *“for me it is subsequent*, *sick*, *trying again*, *sick again*, *trying again*, *sick again”*. This had led to long-term sickness absence from his own job and working in an adjusted job. In a case of breast cancer: *“I had breast cancer twice*, *and was out of work for half a year*, *in between I had several reconstructions and follow-up operations”*.

Besides job demands, some participants mentioned a combination of job and home demands as determinants of frequent sickness absence: *“the combination of having to push yourself to the limit at work and something going on at home”* or *“bad atmosphere at work combined with a busy private life”*, *“the combination of a family and work is more demanding*, *society has become more demanding*. *I have various additional functions*. *Nowadays*, *I really have to plan for a weekend off”*. Participants also mentioned that young women would be inclined to report sick when having sick children at home.

Some participants lamented that low job resources, particularly low social support at the work place (e.g., *“my manager shows no understanding”*, *“problems with your manager”*) caused negative emotions and distance from the work place, resulting in more frequent sickness absence. Two groups discussed this extensively, including their inability to change the situation. One participant even stated that his last sick-leave was a protest: *“the management style makes it easier for me to call-in sick*, *the last time I reported sick was a silent protest”*. He was thinking as follows: *“because I am fed up with the leadership style at my work*, *then I thought*, *when you (management) think to do it that way*, *then tomorrow I am sick for a day”*. He had tried previously to get issues discussed. He stated that you have to be self-critical and expressed a need for taking responsibility (to get issues discussed). When this was not been successful, he called-in sick as a silent protest. It was a compromise at that moment: *“from my heart I would say*, *quit your job*, *leave such management*, *on the other hand*, *I know I will get other problems*: *financially or having difficulty finding another job”* (close to home). Another participant had had collisions with his HRM manager the previous year: *“I didn’t like it at all; possibly that makes that I call in sick more easily when I feel sick and have to vomit”*. When asked for the relationship between bullying, rivalry, divide and conquer policy and frequent sickness absence, we got a literal statement on lowering barriers: *“the barrier*, *the responsibility that you have at first*, *decreases a lot”*. Others also mentioned changed personal attitudes in response to low job resources as a determinant of frequent sickness absence: *“I had a high feeling of responsibility*, *but that has changed; I now feel less committed to my organization”*, *“it is less difficult to call in sick when you feel less social responsibility”*. These changes in personal attitudes had taken place in response to issues at work that the employees could not influence.

In contrast to job resources, neither a lack of home nor personal resources were mentioned as reasons for frequent sickness absence.

Apart from demands and resources, lifestyle and health were mentioned as determinants of frequent sickness absence: *“I have a low back problem due to a lack of exercise… I am frequently ill because of too little exercise”*, *“some people at my work use alcohol and are frequently absent on Monday mornings”* or *“I have colleagues who would be absent less frequently when they lived a healthier and more regular life”*.

Furthermore, chronic health problems were stated as a reason for frequent sickness absence. Some participants suffered from arthrosis, asthma, breast cancer or medically unexplained symptoms. Migraine and liability to catch things were also mentioned.

### Solutions to frequent sickness absence

Some participants had taken action to improve their health and prevent frequent sickness absence, for example by reducing job demands (e.g., *“I work less”*, *“I quit shift work to get better sleep at night”*, “*I currently have accommodated work”*). In all focus groups participants expressed a wish for more job resources, getting more support and feedback from the manager or colleagues: *“respect from manager”*, *“openness amongst colleagues*, *then I would be less concerned about people gossiping behind my back”*, *“a pat on the back helps”*. A clear reference was made to decision latitude and to resources that form a barrier to call in sick: *“feeling appreciated can change the balance between staying at home or going to work”*. One person was preparing a change of jobs because of disrespectful behavior on the part of colleagues: *“I am doing a study to be able to get another job in the future”*. Some participants saw possibilities to improve job resources: *“you have to put things forward for discussion”*, *“support from your boss is important*, *but you have to help the boss in order to get that support”*. Higher job resources were thought important for improving personal resources and vice versa: *“looking for solutions on how to make things work is easier when you are approached in a positive way; this makes you want to work”*.

The participants stated that communication (skills) help to reduce frequent sickness absence: *“I arranged a meeting with my manager (to change shift work)”*, *“learning communication techniques from a company social worker has helped me to focus on solutions in discussions with my manager”*, *“frequent sickness absence conversations may be a good way to trigger people”*. Some participants valued home resources to reduce frequent sickness absence: *“my partner helps me to finish the working week and come back home (also psychologically)”*.

Focus group participants had mixed opinions about the importance of changing lifestyle. Some thought that improving lifestyle would reduce the frequency of sickness absence: *“I have to do sports to clear my head*, *wearing myself out*, *that works*, *then you feel happy*, *then you get energy to go on”*, *“not going out every weekend; when I have had many parties in a weekend everything goes more slowly”*, *“you build reserves when you live in a relaxed way*, *also in your private life”*, *“when something happens*, *you start living more healthily*, *in order to get stronger”*, or *“I am convinced that healthy food helps preventing illnesses”*. Others stated that lifestyle changes would not reduce frequent sickness absence: *“a good lifestyle doesn’t necessarily prevent a chronic disease”*, *“lifestyle doesn’t prevent frequent absences because of migraine”*, *“since I quit smoking*, *I am sick more often*, *despite the idea that to stop smoking and exercise more frequently would increase my physical condition and improve my health”*. Most focus group participants wanted control over their medical situation and sought help: *“I want to look for adequate help myself”*, *“I would like to get help when self-management fails”*. Some mentioned current medical and psychological support as solutions to reduce frequent sickness absence: *“I looked for psychological help”*, *“the last time I visited the general practitioner*, *I urged him to help me and he sent me to a good internist”*, *“an occupational physician helped me to structure things very clearly*, *this helped me a lot”*, *“recognition and acknowledgment from a specialized clinic helped me a lot”*. Some participants felt frustrated when medical doctors could not make a clear diagnosis and saw no treatment opportunities.

In response to the statement ‘I would like to be absent less frequently’, some participants reported that they were not interested in reducing their sickness absence frequency: *“I doubt whether it is necessary to reduce my sickness absence frequency”*, *“I am already infrequently on sick-leave”*. Other participants considered frequent sickness absence as a pattern they would like to change. Many wished to feel healthier: *“I would like to be on sick-leave less frequently because that would mean I felt better”*. However, the statement that frequent absentees might not take good care of themselves went too far: *“you do not report sick on purpose”*, *“you can’t manage your health”*, *“I do sports and eat healthily*, *but still I often have to call in sick”*. There was, however, some room for doubt: *“an accident happens*, *but you can influence your lifestyle”*.

## Discussion

This focus group study showed that many participants were not aware of their sickness absence frequency and almost none of them believed they were at risk of long-term sickness absence in coming years. Frequent absentees mentioned high work and home demands and low job resources as determinants of frequent sickness absence. High job demands led to frequent sickness absence when they surpassed a subject’s capacity. Low job resources led to a reduced feeling of responsibility for work, lowering the barrier to report sick. Additionally, an unhealthy lifestyle, poor health, and chronic illness were regarded as determinants of frequent absence. Although participants put various reasons for frequent absence outside themselves, they wanted to take a role in seeking solutions for the problem. Possibilities for prevention of frequent sickness absence were sought in reducing job demands, increasing job resources, fair communication with the manager and colleagues, better balance between job and home demands, and improvement in health status and lifestyle (albeit not unequivocal). In settings where sickness absence management was poor, employees felt no need to do something about their frequent absence, although they did wish to improve their health.

### Determinants of frequent sickness absence and solutions

The focus group participants attributed their frequent sickness absence to both high job demands and low job resources. Previously, Notenbomer et al. (2015) reported a relationship between frequent sickness absence and work ability in relation to the demands of the job; this also supports a relationship between job demands and frequent sickness absence [23]. Schaufeli et al. (2009), however, reported that low job resources, rather than high job demands were associated with more frequent sickness absence [4]. Possibly, the different results concerning job demands can be explained by differences in study design (qualitative versus quantitative) and study population: Schaufeli and colleagues investigated managers, whereas the participants in our study were employed in various jobs, with other kinds of demands than those of Schaufeli’s managers. They investigated only workload, emotional demands and work–home interference as job demands, while participants in our study also mentioned shift work or climate problems as job demands. Some of our participants also mentioned physical demands like standing for a long time.

Some participants, in dialogue with their managers, had already taken steps to reduce job demands by making accommodations in their work. They were more optimistic about their future at work and about future sickness absence than those who were still struggling with job demands and saw no way out of their work situation. Failure to reduce job demands may be due to lack of personal resources which are known to attenuate job demands[18], work situations that are difficult to solve, inadequate coping strategies or poor skills in solving problems.

Our findings corroborate the relationship between low job resources and frequent sickness absence. We found that frequent absentees reported low support, especially from management, as an important determinant of frequent sickness absence; this is in line with the results of Schaufeli et al. Niedhammer et al. also found that low levels of support at work increased the number of spells, albeit only in men [24]. Low managerial and co-worker support reduced the barriers to report sick; this is in line with the JD-R model, which stipulates that decreasing job resources may reduce an employee’s motivation for work[4]. Participants in our study mentioned a change in their personal attitude in response to low job resources where they used to feel more responsibility. A change in personal attitude seems, however, to be reversible: participants stated that appreciation from a manager would help them to decide to come to work even when they did not feel very well. Here lie possibilities for effective interventions by managers. This is especially important, as many focus group participants found it difficult to increase job resources themselves, and concluded that communication (skill) was required to improve job resources. As both job demands and job resources can underlie frequent sickness absence, managers need more in-depth discussion about job demands in relation to work capacity and potential low job resources. A personal talk between a manager and employee with the JD-R model in mind can lead to more fitting solutions, such as increasing job resources, adapting job demands to remaining work capacity or possibilities to increase work capacity in future. Addressing job resources in team meetings can also help.

Participants saw a link between lifestyle, health and frequent sickness absence, but they found it difficult to change or to continue a changed lifestyle, even when they were interested in improving their health and felt beneficial effects from such changes. Most participants had given up efforts to change their lifestyle,

Many participants when asked about awareness of their high sickness absence frequency focused on the medical reasons for their symptoms. They had been looking for a cure, reduction of or explanation for their complaints. If a cure was not possible, they at least needed to know that they had been thoroughly checked and to be assured that nothing was seriously wrong with them. Some participants felt better merely because medical doctors were understanding and listened to their story. Others were frustrated when doctors did not understand or could not help them. This finding is relevant for physicians. They could take complaints seriously, without medicalizing symptoms and signs. Previous studies have proven that there is a relationship between frequent sickness absence and poor health [6, 25], underpinning the feeling of participants that they need a thorough check. Roskes et al. (2005) reported that patients with a chronic illness had more frequent sickness absences [26]. Our participants also thought that a chronic condition was a determinant of frequent sickness absence. This emphasizes that poor health may not only be the result of a mismatch between demands and work capacity, but in itself is a cause of frequent sickness absence. Medical reasons should therefore not only be seen as potential justifications for frequent sickness absence, but should in themselves be taken seriously. An occupational physician can help to determine the influence of medical reasons on frequent sickness absence and check if suitable medical help has been established.

### Study strengths and weaknesses

To our knowledge, this is the first focus group study to explore the ideas and thoughts of frequent absentees about their sickness absence. We recruited employees from various economic sectors and occupations and ensured the privacy and confidentiality of group discussions by including only participants employed at different companies. Because the focus group moderator was new to the subject and to the participants, group discussions were unprejudiced and open. The moderator ensured contribution by all participants by starting with an introduction round to accustom all participants to talking in the group, and by asking for opinions and ideas when a participant did not spontaneously join the discussion.

The participants came from organizations staffing >100 employees in the province of Friesland. Employees working in larger organizations might have different views on frequent sickness absence than those working in small businesses where sickness absence threatens staffing levels and, therefore putting greater pressure on employees to attend. Thus, we may not have heard all possible reasons for frequent sickness absence. However, the aim of this qualitative study was to gain insight into the thoughts and beliefs of frequent absentees and the frequent sickness absence itself instead of finding general characteristics that apply to the workforce.

The results of this study should be validated by further quantitative research in larger working populations. It would be interesting to investigate whether increased awareness of frequent sickness absence reduces its frequency as well as the risk of future long-term sickness absence. Further research on the effects of both job and home demands and resources as well as personal resources could provide clues for interventions to reduce or prevent frequent sickness absence.

## Conclusions

The JD–R model provided a framework for determinants of frequent sickness absence and solutions to reduce its frequency. Additional determinants that did not fit into the JD-R model were poor health, chronic illness, unhealthy lifestyles, and diminished personal attitudes. Reduction or accommodation of job demands and improvement of job resources were considered solutions to reduce frequent sickness absence. Focus group participants thought that improvement of job resources would be difficult and require good communication skills on the part of employees. It would be the task of managers to raise this issue in team briefings and personal talks.

We propose that frequent sickness absence should be regarded as a signal that something is wrong. Managers, supervisors, and occupational health providers should advise and support frequent absentees to accommodate job demands, improve job and personal resources, and improve health rather than express disapproval of frequent sickness absence and apply pressure on frequent absentees regarding work attendance.
